# Effectiveness and Safety of Real-Time Transthoracic Ultrasound-Guided Thoracentesis

**DOI:** 10.3390/diagnostics12030725

**Published:** 2022-03-16

**Authors:** Marco Sperandeo, Carla Maria Irene Quarato, Rosario Squatrito, Paolo Fuso, Lucia Dimitri, Anna Simeone, Stefano Notarangelo, Donato Lacedonia

**Affiliations:** 1 Unit of Interventional and Diagnostic Ultrasound of Internal Medicine, Istituto di Ricovero e Cura a Carattere Scientifico (IRCCS) “Fondazione Casa Sollievo della Sofferenza”, 71013 San Giovanni Rotondo, Italy; sperandeomar@gmail.com; 2 Department of Medical and Surgical Sciences, Institute of Respiratory Diseases, Policlinico Universitario “Riuniti” di Foggia, University of Foggia, 71121 Foggia, Italy; paolo.fuso91@gmail.com (P.F.); donato.lacedonia@unifg.it (D.L.); 3 Azienda Sanitaria Provinciale di Catania, 95125 Catania, Italy; rosario.squatrito@gmail.com; 4 Unit of Patology, Istituto di Ricovero e Cura a Carattere Scientifico (IRCCS) “Fondazione Casa Sollievo della Sofferenza”, 71013 San Giovanni Rotondo, Italy; lmcdimitri@libero.it; 5 Unit of Radiology, Istituto di Ricovero e Cura a Carattere Scientifico (IRCCS) “Fondazione Casa Sollievo della Sofferenza”, 71013 San Giovanni Rotondo, Italy; a.simeone@operapadrepio.it; 6 Pneumologia, Azienda Sanitaria Locale (ASL) di Foggia, 71121 Foggia, Italy; stenotar@gmail.com

**Keywords:** transthoracic ultrasound, pleural effusion, transthoracic ultrasound-guided thoracentesis, effectiveness, safety

## Abstract

Purpose: The purpose of the present study was to specifically evaluate the effectiveness and safety of real-time ultrasound-guided thoracentesis in a case series of pleural effusion. Patients and methods: An observational prospective study was conducted. From February 2018 to December 2019, a total of 361 consecutive real-time transthoracic ultrasound (TUS)-guided thoracentesis were performed in the Unit of Diagnostic and Interventional Ultrasound of the Research Hospital “Fondazione Casa Sollievo della Sofferenza” of San Giovanni Rotondo, Foggia, Italy. The primary indication for thoracentesis was therapeutic in all the cases (i.e., evacuation of persistent small/moderate pleural effusions to avoid super-infection; drainage of symptomatic moderate/massive effusions). For completeness, further diagnostic investigations (including chemical, microbiological, and cytological analysis) were conducted. All the procedures were performed by two internists with more than 30 years of experience in interventional ultrasound using a multifrequency convex probe (3–8 MHz). For pleural effusions with a depth of 2–3 cm measured at the level of the costo-phrenic sinus was employed a dedicated holed convex-array probe (5 MHz). Results: In all the cases, the attempts at thoracentesis were successful, allowing the achievement of the therapeutic purpose of the procedure (i.e., the complete drying of the pleural space or the withdrawal of fluid till a “safe” quantity [a mean of 1.5 L, max 2 L] producing relief from symptoms) regardless of the initial extent of the pleural effusion. There were only 3 cases of pneumothorax, for a prevalence rate of complications in this population of 0.83%. No statistical difference was recorded in the rate of pneumothorax according to the initial amount of pleural fluid in the effusion (*p* = 0.12). All the pleural effusions classified as transudates showed an anechoic TUS appearance. Only the exudative effusions showed a complex nonseptated or a hyperechoic TUS appearance. However, an anechoic TUS pattern was not unequivocally associated with transudates. Some chronic transudates have been classified as exudates by Light’s criteria, showing also a complex nonseptated TUS appearance. The cytological examination of the drained fluid allowed the detection of neoplastic cells in 15.89% cases. On the other hand, the microbiological examination of effusions yielded negative results in all the cases. Conclusions: Real-time TUS-guided thoracentesis is a therapeutically effective and safe procedure, despite the diagnostic yield of the cytological or microbiological examinations on the collected liquid being very low. Future blinded randomized studies are required to definitely clarify the actual benefit of the real-time TUS-guided procedure over percussion-guided and other ultrasound-based procedures.

## 1. Introduction

Pleural effusion represents a common condition among patients hospitalized in internal medicine and pneumology departments [1]. The most frequently associated causes are congestive heart failure, bacterial pneumonia, viral diseases, pulmonary embolism, and malignancy [2,3]. Symptoms are non-specific and often indistinguishable from those of the underlining disease process, including cough, dyspnea and pleuritic chest pain.

In the case of pleural effusion, a thoracentesis can be performed for both diagnostic and therapeutic purposes. Sampling of pleural fluid through percutaneous thoracentesis is diagnostically performed to macroscopically visualize and microscopically characterize the effusion by analyzing its chemical, microbiological, and cellular content [4]. This allows to determine its nature (i.e., transudate, exudate) and to identify potential causes. On the other side, therapeutic thoracentesis is performed with the goal to prompt symptomatic relief and restoration of quality of life to patients suffering from related dyspnea or to avoid the superinfection of chronic effusions [5].

A chest radiograph is usually the first-line examination used to assess for the presence of pleural effusion. However, at least 50 mL of fluid must accumulate in costophrenic recesses for a lateral upright chest radiography to suggest the presence of a pleural effusion. On the other hand, on a standard posterior-anterior chest radiograph, blunting of the costophrenic recesses and obliteration of the hemidiaphragm are seen only when more than 200–500 mL of pleural fluid has accumulated [6]. Furthermore, a supine anterior-posterior chest radiograph can miss even a significant proportion of large effusion [7].

Transthoracic ultrasound (TUS) is an imaging method presenting several advantages, such as no radiation exposure, cost-containment, easy transportability allowing bedside use, noninvasiveness, and prompt repeatability when necessary. In recent years, there has been an increasing interest in the use of TUS for the evaluation of pleuro-pulmonary diseases; moreover, ultrasound has developed as an important tool in the hands of pulmonologists and internists. In particular, TUS is traditionally considered the “gold standard” method for the study of pleural effusion [8,9]. The identification of pleural effusion on TUS is also possible for an amount of liquid below 10 mL, and therefore, with a diagnostic yield far superior to standard chest radiography [9]. In the case of a white hemithorax on chest X-rays, TUS allows the distinction between effusion and lung consolidations, confirming or not the diagnosis of massive effusion. The aspect of pleural effusion on TUS can suggest the nature of the fluid, although a definitive diagnosis requires a thoracentesis in order to allow physical, chemical, and microbiological studies. According to the characteristics of the pleural effusion on ultrasound, it can appear as anechoic (black), complex nonseptated (black with white strands), complex septated (black with white septa), or homogeneously echogenic (white). In general, the presence of complex pleural effusion suggests exudative effusion, whereas an anechogenic effusion might be transudative. However, exudates too can be visualized as an anechoic area [10,11].

The use of TUS in performing a thoracentesis reduces the rate of complications when compared with the traditional percussion method. As reported elsewhere, the percentage of pneumothorax without the assistance of ultrasound is estimated from 8.89 to 10.3%, which falls to 0.97–4.9% if assisted by ultrasound [12,13,14]. TUS allows the identification of the best site to perform the procedure and the measurement of the depth of the adjacent organs in order to avoid organ injury during puncture. As a result, the BTS guidelines strongly recommend using TUS before procedures involving pleural effusion [15]. Since TUS allows to make a diagnosis of the nature of the effusion, it is possible to decide in advance which gauge needle must be used (for example, a 20 G needle if the effusion is anechoic, while a corpusculate effusion could require the use of a larger 18 G needle) [16].

TUS may be employed in two different ways when performing a thoracentesis: (1) as a landmark method to identify the best site of the puncture; (2) as a real-time guide to closely monitor the entire procedure by continuous visualization of the needle. Not all the studies relating to the use of ultrasound in thoracentesis specify the actual role of the method in assisting the procedure.

The aim of this observational study was to specifically evaluate the effectiveness and safety of real-time ultrasound-guided thoracentesis in a case series of pleural effusion.

## 2. Materials and Methods

This was a prospective single-center observational study. From February 2018 to December 2019, 361 consecutive real-time ultrasound-guided thoracenteses were performed in the Unit of Diagnostic and Interventional Ultrasound of the Research Hospital “Fondazione Casa Sollievo della Sofferenza” of San Giovanni Rotondo, Foggia, Italy. The study followed the amended Declaration of Helsinki and the local institutional Ethical Review Board approved the protocol (TACE-CSS, n 106/2018).

The primary endpoint of our study was to analyze the effectiveness and safety of real-time ultrasound-guided thoracentesis in a case series of pleural effusion. Pleural effusion was diagnosed by chest X-ray, TUS, or chest computed tomography (CT) according to the clinical applicability for each patient. If the attending physician judged the patient to be a candidate for drainage, a TUS assessment was performed by an internist with more than 30 years of experience in diagnostic and interventional ultrasound to confirm the presence of pleural effusion and to determine whether it was susceptible to drainage. For all the patients, the primary indication for thoracentesis was therapeutic, including the following: (1) the evacuation of small/moderate pleural effusions that persisted for more than 3 days in order to prevent the development of an infection of the pleural space; (2) the drainage of moderate/massive effusions in order to obtain relief from symptoms. The “effectiveness” of the procedure was judged in terms of technical success in withdrawing a quantity of liquid suitable for therapeutic purposes (i.e., the complete drying of the pleural space or the aspiration of fluid until a “safe” quantity was capable of producing relief from symptoms). The “safety” was assessed in terms of complication rate.

For completeness, further diagnostic investigations, including chemical, microbiological, and cytological analysis, were conducted using the first 30 mL of withdrawn liquid. The ultrasound findings of the effusion were then correlated to its macroscopic appearance and to the results of the chemical-physical examination (i.e., transudate or exudate) and the diagnostic yield of the cytological and microbiological tests was finally calculated.

Exclusion criteria were: (1) pleural effusion height measuring less than 10 mm in the costophrenic sinus; (2) a prolonged prothrombin time (PT-INR > 1.5) or a platelet count < 50,000; (3) severe renal failure (serum creatinine > 6 mg/dL); (4) positive pressure ventilation; (5) skin infection at the collection site. All the participants provided informed written consent for the procedure.

### 2.1. Transthoracic Ultrasound (TUS) Examination

All patients received TUS scanning for diagnosis, for estimation of effusion characteristics (i.e., anechoic, complex nonseptated, complex septated, or homogeneously echogenic), and for fluid quantification (in mm). TUS examination was performed by an Esaote MyLab-9 scanner (Esaote-Biomedica, Genoa, Italy) using a convex multi-frequency probe (3–8 MHz). The detailed machine settings for US imaging acquisition were as follows: depth varying between 70–140 mm, time gain compensation (TGC) no more than 50%, focus pointed at the hyperechoic pleural line, activation of the tissue harmonic imaging. Patients were examined in a sitting or semi-sitting position, exploring the chest from the bottom (highlighting the acoustic windows of the liver on the right and the spleen on the left and therefore starting from the identification of the respective costo-diaphragmatic sinuses) to the top, with longitudinal intercostal scans, along the paravertebral and hemiscapular lines, posteriorly, along the postero-axillary, mid-axillary and anterior-axillary lines, laterally, and along the hemi-clavicular and parasternal line, anteriorly. Once the pleural effusion was clearly visualized in longitudinal position at the lung base in the posterior axillary line, the transducer was then rotated to obtain a transverse view and the intra-pleural distance of the effusion was measured. The amount of the pleural fluid was semi-quantitatively classified as “small” if the fluid collection was viewable at the level of a single intercostal space; as “moderate” if the fluid collection was appreciated at the level of two or three intercostal spaces and as “large” if the fluid collection extended for more than three intercostal spaces from costophrenic angle [17,18]. The presence or absence of the pleural “gliding or sliding sign” (i.e., the visible real-time movement of coming and going of the hyperechoic (white) pleural line with respiratory excursions [8,9]) was also evaluated before and after the thoracentesis procedure.

### 2.2. Thoracentesis

**Figure 1:**
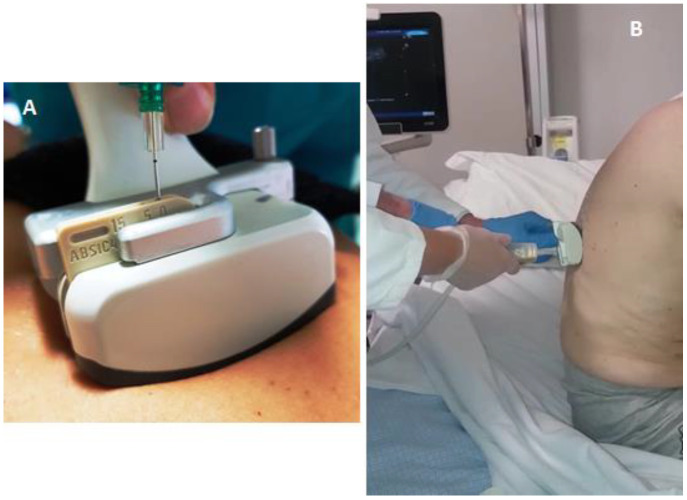
(**A**) Dedicated convex-array probe (5 MHz) equipped with a holed guide for needle insertion during interventional procedures. (**B**) Ultrasound-guided thoracentesis performed by the employment of a dedicated holed convex-array with the patient in a sitting position.

All thoracenteses were performed by two internists with more than 30 years of experience in interventional ultrasound. The procedure has been carried out with was employed the same ultrasound scanner used for TUS examination (Esaote MyLab-9, Esaote-Biomedica, Genoa, Italy) and a multifrequency convex-array probe (3–8 MHz). For pleural effusions with a depth of 2–3 cm measured at the level of the costo-phrenic sinus (in 12 patients), a dedicated convex-array probe (5 MHz) equipped with a holed guide was employed for needle insertion during interventional procedures (Figure 1).

Patients were placed in a sitting position, leaning forward with arms resting on a bedside table, or in a semi-sitting position with the head of the bed elevated to 30 or 45 degrees and the patient’s arm on the affected side above his head. Once the pleural effusion was clearly visualized on B-mode TUS, the needle insertion point was selected in the posterior axillary line at the upper border of the rib, one intercostal space below the top of the effusion. Sterilization of the site was obtained by applying a 1% solution of povidone-iodine. An 18 G needle was attached to a 3-way stopcock, placing a 50 mL syringe on one port of the stopcock and attaching a drainage tube to the other port. For smaller effusion, the use of a 20 G needle was preferred. The needle was inserted along the upper border of the lower rib in the chosen inter-costal space and advanced into the effusion while aspirating. Thirty milliliters of fluid was withdrawn into the syringe and placed in appropriate tubes and bottles for testing. For evacuative thoracentesis, the additional liquid was slowly aspirated in the syringe and injected back into the drainage tube, appropriately using the 3-way stopcock system. The needle puncture was performed over the center of the transducer by visualizing in real time needle insertion and lung re-expansion during drainage the entire time. No more than 2 L of fluid were removed in order to reduce the risk of post-expansion pulmonary edema. The procedure was, in any case, stopped if the patient experienced cough, dyspnea, or chest pain. After the procedure, TUS was used to rule out the presence of pneumothorax in patients who presented the pleural “gliding or sliding sign” before the thoracentesis. A chest X-ray performed within 24 h from the procedure was also reviewed.

### 2.3. Statistical Analysis

Results were expressed as mean ± standard deviations (SD) for numerical data and as number (n) and percentage (%) for categorical data. Macroscopic, microscopic, and TUS descriptions of the pleural fluid and procedure’s complications were analyzed. The pleural fluid was classified as an exudate if one or more of the following Light’s criteria [19,20] were met: (1) pleural fluid protein/serum protein ratio > 0.5; (2) pleural fluid LDH/serum LDH ratio > 0.6; pleural fluid LDH more than two thirds the upper limit of normal serum LDH (i.e., >200 IU). The difference in TUS appearance between transudates and exudatives was calculated using the ANOVA and Student’s *t*-test. The sub-analysis in terms of the rate of complications in small, moderate, and large effusions was performed using the ANOVA test. A *p* less than 0.05 was considered significant.

## 3. Results

A total of 361 real-time TUS-guided thoracenteses were performed with the described technique. Moreover, 69.3% of the patients were men and 30.7% were women; the mean age of the observed group was 64 ± 8 years. In 100% of cases, the indication for thoracentesis was therapeutic. In fact, the main indication consisted in the evacuation of small/moderate pleural effusions that persisted for more than 3 days or in the drainage of moderate/massive effusions causing dyspnoea. Further diagnostic investigations on the pleural fluid (which included chemical and microbiological studies, as well as cytological analysis) were carried out in all cases as part of a standard routine protocol.

### 3.1. Technical Success (“Effectiveness”) of the Procedure

In all the cases, the attempts at thoracentesis were successful. A mean of about 1.5 L (max 2 L) of fluid was removed. Based on the amount of fluid collection assessed by the method of counting of intercostal spaces, 23 patients (6.37%) had a small pleural effusion, 246 patients (68.14%) had a moderate pleural effusion, and 92 patients (25.49%) had a large pleural effusion. The actual volume of fluid drained consisted of about 500 mL in small effusions, 1000–1500 mL in moderate effusions, and 1500–2000 mL in large effusions. The therapeutic purpose was achieved in all the cases with the complete drying of the pleural space or the withdrawal of fluid till a “safe” quantity that was capable of producing relief from symptoms.

### 3.2. TUS Findings versus Macroscopic Aspect/Chemical-Physical Analysis of Pleural Effusions and Diagnostic Results

Following the clinical-radiological evaluation and/or the histological examination of any lesions associated with the effusion, the final diagnoses presented by the enrolled patients were: heart failure in 33 cases (9.14%), pneumonia in 114 cases (31.58%), primary lung cancer in 202 cases (55.96%), and lung metastasis in 12 cases (3.32%).

**Figure 2:**
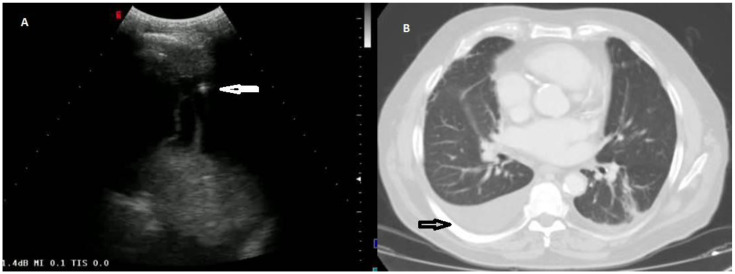
(**A**) TUS scan showing an anechoic effusion with consensual parenchymal atelectasis during thoracentesis with a multifrequency convex probe (3.5 MHz). The tip of the needle is highlighted by a white arrow. (**B**) The corresponding CT scan shows a smooth thickening of the peribronchovascular interstitium and a bilateral pleural effusion with passive atelectasis of lower lobe in the right lung (black arrow).

According to the macroscopic appearance, in 54 patients (14.96%) pleural effusion was hemorrhagic, in 297 (82.27%) citrine, and in 10 patients (2.77%) torbid. Based on the composition of the drained fluid, pleural effusions were classified as transudate in 27 patients (7.48%) and exudates in 334 patients (92.52%). On TUS examination, pleural effusions showed an anechoic appearance in 165 patients (45.71%), a heterogeneous ipo-iperechoic appearance (i.e., complex nonseptated) in 174 patients (48.20%), and a homogeneous hyperechoic appearance in 22 patients (6.09%). All the pleural effusion (100.0%) classified as transudates according to Light’s criteria showed an anechoic TUS appearance (Figure 2).

**Figure 3:**
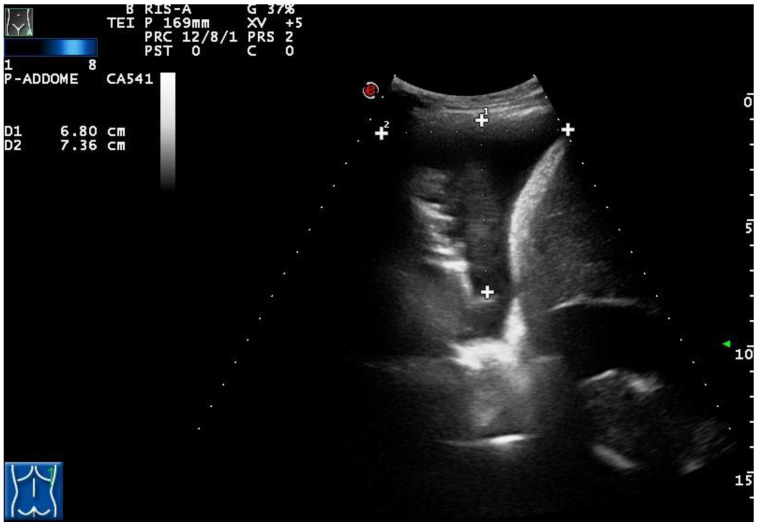
A complex nonseptated pleural exudate in a patient with pneumonia, measured by two orthogonal views (longitudinal and transversal), viewed by longitudinal scan using a convex multifrequency probe (3.5 MHz).

Only the exudative effusions showed a complex nonseptated or a hyperechoic TUS appearance. Considering the pleural effusions classified as exudates, the frequency distribution of the anechoic TUS appearance (n = 138/334, 41.32%) and of the complex nonseptated pattern (n = 174/334, 52.09%) were statistically higher than that of the homogeneous hyperechoic one (22/334, 6.59%), with a *p*-value < 0.0001. The number of complex nonseptated exudates was statistically higher compared to that of the anechoic ones (*p* = 0.005) (Figure 3).

**Figure 4:**
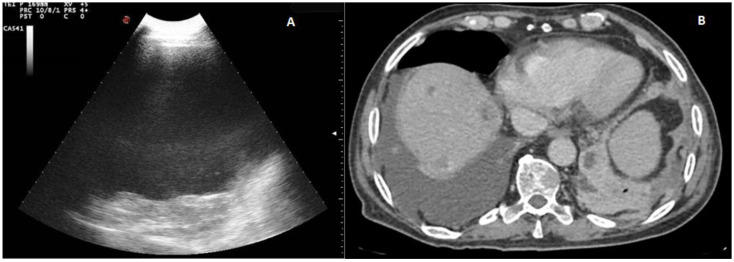
(**A**) TUS scan showing a homogeneous iperechoic pleural exudates viewed by a convex multifrequency probe (3.5 MHz). The drained fluid was macroscopically hemorrhagic. (**B**) The corresponding CT scan shows a large right effusion in a patient with a diagnosis of metastatic kidney cancer.

Twenty out of 22 homogeneous hyperechoic effusions (90.91%) were macroscopically hemorrhagic (Figure 4).

**Figure 5:**
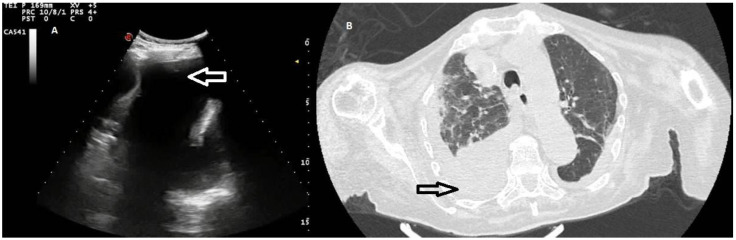
(**A**) TUS scan showing a complex nonseptated effusion with consensual parenchymal atelectasis. The tip of the needle during TUS-guided thoracentesis with a multifrequency convex probe (3.5 MHz) is highlighted by a white arrow. (**B**) The corresponding CT scan shows an extensive apico-parieto-basal pleural effusion of greater right expression (black arrow) with consensual lower lobe atelectasis. A bilateral thickening of interlobular septa and some right ground-glass opacities with partial sparing of the lung periphery are also present (congestive heart failure).

Six of the 33 effusions (18.18%) presented by patients diagnosed with heart failure were classified as exudates. Such effusions presented a complex nonseptated appearance on TUS examination (Figure 5).

Table 1 resumes macroscopic, microscopic and TUS description of the pleural fluid in each diagnosed clinical condition.

The cytological examination of the drained fluid allowed the detection of neoplastic cells in 34/214 cases (15.89%). Among them, 28 patients received a diagnosis of lung adenocarcinoma, while the remaining 6 patients had a diagnosis of lung metastasis. The microbiological investigation of the effusion yielded a negative result in all the cases of pneumonia.

### 3.3. Safety of the Procedure

No severe complications were observed. Only 3 patients (0.83%) had an iatrogenic partial pneumothorax (among which 1 case was a hydropneumothorax) with spontaneous full lung re-expansion without the need for chest tube drainage. More specifically, one case of pneumothorax was recorded in a patient with a small effusion, while the remaining cases of pneumothorax and hydropneumothorax occurred in two patients with moderate effusion (no cases occurred in patients with massive effusion). As a result, there was no statistical difference in the rate of pneumothorax according to the initial amount of pleural fluid in the effusion (*p* = 0.12).

The presence of the “sliding or gliding sign” was documented in 320 patients (88.64%) before the thoracentesis procedure was performed. After the procedure, in those 2 patients who developed an iatrogenic pneumothorax, the “sliding or gliding sign” was no longer present, while, in that patient who had a hydropneumothorax, TUS assessed the presence of the “curtain sign”, a dynamic overlapping ultrasound artifact resulting from the presence of free air within the pleural effusion [21]. In the remaining 41 patients (11.36%), the “sliding or gliding sign” was absent also before thoracentesis, so it was not possible to evaluate it after the procedure as a sign of possible pneumothorax. TUS examination in these patients revealed a complex nonseptated effusion.

Seven patients had a persistent cough during the course or at the end of the pulmonary rehabilitation during thoracentesis, with no statistical difference based on the initial amount of pleural fluid (i.e., 1 case in a patient with small effusion, 3 cases in patients with moderate effusions, and 3 cases in patients with massive effusions, *p* = 0.33).

No patients showed post-procedural chest hematoma, chest pain, or hemorrhage, although the laceration of an intercostals neurovascular bundle with the subsequent development of a hemothorax is another potential complication following thoracentesis.

## 4. Discussion

Based on the results of the present study, real-time ultrasound-guided thoracentesis was confirmed to be a technically effective procedure, which allowed the evacuation of a sufficient amount of fluid for therapeutic purposes regardless of the initial extent of the pleural effusion. Although the diagnostic yield of cytological examination performed on pleural fluid was very low, with diagnostic results of microbiological texts resulting even null, ultrasound-guided thoracentesis configured a safe procedure, with a prevalence rate of pneumothorax of only 0.83%.

Not all patients with pleural effusion should undergo thoracentesis. For example, in patients with a clinically evident heart failure, the procedure is not indicated with the exception of a case of massive effusion causing severe dyspnea, the lack of resolution with effective diuretic therapy, an echocardiogram that is inconsistent with heart failure, signs of infection and fever or features suggestive for an alternative etiology of the effusion (i.e., bilateral effusions of significantly disparate sizes) [22]. Pneumonia is associated with an exudative pleural effusion in up to 57% of cases. Resolution is usually obtained with antibiotic treatment, but a certain number of effusions will progress to an infected pleural space, implying the indication for fluid drainage [5,23,24]. The cause of any dyspnea in cancer patients must be investigated by TUS, which, in the case of finding a massive pleural effusion, may easily guide a prompt evacuative drainage [25]. Typically, in patients with lung cancer, the cytological examination of the eventually associated pleural effusion may represent the first approach to make a diagnosis. Furthermore, as a therapeutic evacuative drainage usually does not prevent an effusion from reforming again and in a short time in patients with lung or pleural cancer, the presence of a mid-basal effusion is usually indicative of inserting a drainage tube under ultrasound guidance [25,26]. In the case of cancer, TUS can also aid in identifying pleural thickening and nodulations that often accompany neoplastic effusions [27]. In our study, all thoracentesis procedures were conducted with therapeutic intent, including the evacuation of small/moderate pleural effusions that persisted for more than 3 days in order to prevent the development of an infection of the pleural space or the drainage of moderate/massive effusions causing dyspnoea. In all the cases, the amount of aspirated fluid allowed the achievement of the therapeutic purpose for which the procedure was performed (i.e., the complete drying of the pleural space or the withdrawal of fluid till a “safe” quantity that was capable, anyway, of producing relief from symptoms).

As part of a standard routine protocol, further diagnostic investigations (including chemical, microbiological, and cytological studies) were carried out on the pleural fluid. According to this protocol, the first step consisted of the analysis of pleural fluid for pleural lactate dehydrogenase (LDH) and proteins in order to establish its exudative or transudative nature according to Light’s criteria [5]. Exudative effusions have a higher protein concentration (>30 g/L) resulting from an increased capillary permeability related to several local inflammatory processes, including pneumonia and cancer. On the other hand, transudative effusions result from imbalances in hydrostatic and oncotic forces and are caused by a limited number of recognized clinical conditions such as heart failure [28,29]. However, a certain percentage of initially transudative effusions can be classified as exudates by Light’s criteria, as diuretic therapy increases the total protein and lactate dehydrogenase concentrations in pleural transudates due to heart failure and chronic transudative effusions naturally tend to form fibrin septa over time [30]. This occurrence was confirmed also in our study, in which 18.18% of the effusions presented by patients diagnosed with heart failure were classified as an exudate. Such effusions also showed a complex nonseptated appearance on TUS examination.

In patients with exudative effusion, further microbiological and cytological analyses on the drained fluid should be performed [5,31,32]. In our experience, a diagnosis of malignant effusion was performed in 34/214 patients (15.89%) by the detection of neoplastic cells at the cytological examination of the drained fluid. The cytological yield was higher for adenocarcinoma, according to what is also reported by the literature [33,34,35]. On the other hand, the microbiological examination of the effusion yielded negative results in all the cases of pneumonia. This result is in line with the usual absence of bacteria in pleural effusions (i.e., sterility) [36].

However, the incidence of post-procedural complications was very low. In particular, the rate of iatrogenic pneumothorax documented in our experience was even lower than that reported in the current literature, ranging from 0.97% to 4.9% [12,13,14,37,38,39]. This optimal result was certainly possible thanks to the support of the ultrasound examination in several stages of the procedure.

**Figure 6:**
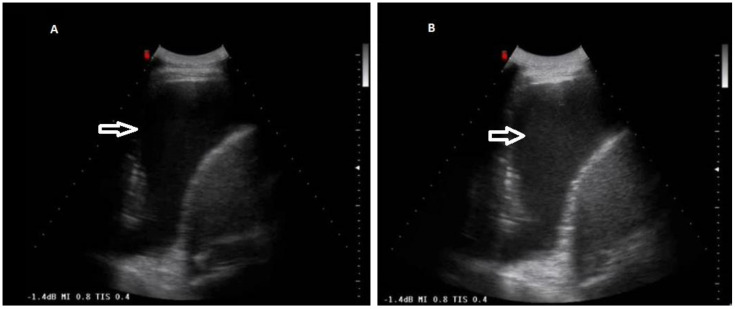
Gain variation on the same TUS scan. (**A**) Anechoic pleural effusion with correct gain setting. (**B**) Falsely hyperechoic pleural effusion due to improper gain increase.

First, the ultrasound made it possible to hypothesize the nature of the effusion and consequently the choice of the most appropriate needle (i.e., 20 G or 18 G). As a confirmation, all the transudates in our experience showed an anechoic TUS appearance, while only the exudative effusions showed a complex nonseptated or hyperechoic TUS appearance [11]. In this regard, it is useful to remember that the appropriate setting of the ultrasound scanner is essential for the correct evaluation of the effusion’s sonographic pattern. In particular, a gain curve adjusted in excess (increased gain) may commute an anechoic pleural effusion in a hyperechoic one [16] (Figure 6).

Second, the ultrasound examination revealed the depth of the effusion and the most appropriate site to perform the thoracentesis.

**Figure 7:**
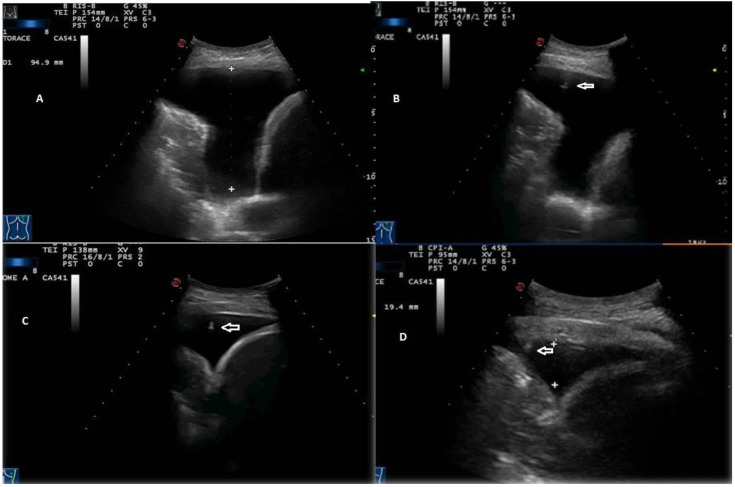
Stages of TUS-assisted thoracentesis. (**A**) Initial pleural effusion’s measurement using a convex 3.5 MHz holed probe. (**B**–**D**) Progressive lung re-expansion during drainage. The position of the needle tip (white arrows) is highlighted during all phases of the procedure until the needle is retracted.

Third, the real-time TUS B-mode scans made it possible to monitor the reduction of the pleural fluid content during drainage, allowing the needle to be retracted as the lung parenchyma returned to the wall and until its removal after complete lung rehabilitation (Figure 7).

In addition, it is noteworthy that for effusions with a depth of 2–3 cm the use of a holed probe, dedicated for TUS-guided interventional procedures, allowed to guide the entire procedure under a co-axial view [40].

Last but not least, the ultrasound study in real time after thoracentesis allows to immediately identify the presence or absence of an iatrogenic pneumothorax in all those patients in whom the “gliding or sliding” sign was present before the procedure with a high positive predictive value. In some cases, as reported in the present experience, TUS may also show the so-called “curtain sign”, an ultrasound artifact described as pathognomonic of hydropneumothorax [21]. Furthermore, 11.36% of patients in our study did not show the gliding sign also before the thoracentesis procedure was performed. These patients had a complex nonseptated effusion, demonstrating that the presence of initial organization of the effusion can limit pleural motion by configuring a false positive of pneumothorax [41]. In such cases, a diagnosis of pneumothorax can be suspected only on the basis of clinical data (e.g., pain and dyspnea onset) and confirmed by a chest X-ray [42,43,44,45].

No patients showed post-procedural hemorrhage resulting from the inadvertent laceration of an intercostal artery, which is another possible complication not avoidable by using ultrasound. The experience of the operator and the choice of a safe lateral site to perform the puncture have certainly played a role in avoiding this occurrence. In addition, the ultrasound finding of a complex or hyperechoic fluid reaccumulation after the procedure should allow early detection, prompting further investigation and intervention.

The strength of the present prospective observational study is that it described a large series of thoracentesis procedures specifically performed under ultrasound guidance in real time, confirming the safety of this technique. The main limitation is that the study does not allow assessing the safety of real-time TUS-guided thoracentesis in comparison with the static landmark TUS-based method or the traditional percussion-based technique. However, several studies comparing physical examination-guided (blind) thoracentesis and ultrasound-guided thoracentesis have shown a lower rate of complications with sonographic guidance [12,13,14]. Furthermore, despite our study not including complex septated effusions, TUS is more sensitive than chest computed tomography (CT) in detecting septations in the pleural fluid [46] and, as reported elsewhere, real-time TUS guidance has the advantage to allow the safe direction of a chest drain placement into the largest locule visualized [5,47].

## 5. Conclusions

The real-time use of TUS before, during, and immediately after thoracentesis is an effective, safe, and easily performing procedure, which limits post-procedural complications in a very excellent way. The TUS-guide reduced the number of unsuccessful attempts at thoracentesis (“dry tap”) to “zero”, which may, instead, occur during the percussion-based technique [48]. The technique allowed the aspiration of a sufficient quantity of liquid for therapeutic purposes from effusions of each entity, although the diagnostic yield of the cytological or microbiological examinations on this liquid was very low. Furthermore, our experience demonstrated a sensible improvement in the rate of iatrogenic pneumothorax, which is the most commonly recognized complication associated with the procedure. In addition, the TUS study, before and after thoracentesis, can also allow to immediately identify the presence or absence of an iatrogenic pneumothorax by ascertaining the disappearance of the “gliding or sliding” sign and to detect, early on, an eventual post-procedural hemothorax by highlighting a complex or hyperechoic fluid reaccumulation after the procedure. The main limitation of this technique may be to lengthen the time required to carry out the procedure. However, this risk is widely acceptable considering the possible benefits in terms of economic resources deriving from the dramatic reduction of all the post-procedural complications including, firstly, the percentage of iatrogenic pneumothorax. Future blinded randomized studies should definitely clarify the actual benefit of the real-time TUS-guided procedure over the percussion-guided and other ultrasound-based procedures.

## Figures and Tables

**Table 1:** Macroscopic, microscopic, and TUS description of the pleural fluid according to the diagnosed clinical condition.

	Heart Failure n = 33	Pneumonia n = 114	Primary Lung Cancer n = 202	Lung Metastasis n = 12	Total n = 361
Hemorrhagic	0 (0.00%)	12 (10.53%)	34 (16.83%)	8 (66.67%)	54 (14.96%)
Citrine	33 (100.00%)	96 (84.21%)	164 (81.19%)	4 (33.33%)	297 (82.27%)
Torbid	0 (0.00%)	6 (5.26%)	4 (1.98%)	0 (0.0%)	10 (2.77%)
Transudate	27 (81.82%)	0 (0.0%)	0 (0.0%)	0 (0.0%)	27 (7.48%)
Exudate	6 (18.18%)	114 (100.0%)	202 (100.0%)	12 (100.0%)	334 (92.52%)
Anechoic	27 (81.18%)	46 (40.35%)	89 (44.06%)	3 (25.00%)	165 (45.71%)
Complex nonseptated	6 (18.18%)	58 (50.88%)	103 (50.99%)	7 (58.33%)	174 (48.20%)
Hyperechoic	0 (0.00%)	10 (8.77%)	10 (4.95%)	2 (16.67%)	22 (6.09%)

## Data Availability

The data presented in this study are available on request from the corresponding author. The data are not publicly available due to ethical reasons.
